# An immunogenic cell death-related regulators classification patterns and immune microenvironment infiltration characterization in intracranial aneurysm based on machine learning

**DOI:** 10.3389/fimmu.2022.1001320

**Published:** 2022-09-29

**Authors:** Mirzat Turhon, Aierpati Maimaiti, Dilmurat Gheyret, Aximujiang Axier, Nizamidingjiang Rexiati, Kaheerman Kadeer, Riqing Su, Zengliang Wang, Xiaohong Chen, Xiaojiang Cheng, Yisen Zhang, Maimaitili Aisha

**Affiliations:** ^1^ Department of Neurointerventional Surgery, Beijing Neurosurgical Institute, Capital Medical University, Beijing, China; ^2^ Department of Neurointerventional Surgery, Beijing Tiantan hospital, Capital Medical University, Beijing, China; ^3^ Department of Neurosurgery, Neurosurgery Centre, The First Affiliated Hospital of Xinjiang Medical University, Urumqi, China

**Keywords:** immunogenic cell death, intracranial aneurysm, immune microenvironment, risk signature, machine learning

## Abstract

**Background:**

Immunogenic Cell Death (ICD) is a novel way to regulate cell death and can sufficiently activate adaptive immune responses. Its role in immunity is still emerging. However, the involvement of ICD in Intracranial Aneurysms (IA) remains unclear. This study aimed to identify biomarkers associated with ICDs and determine the relationship between them and the immune microenvironment during the onset and progression of IA

**Methods:**

The IA gene expression profiles were obtained from the Gene Expression Omnibus (GEO) database. The differentially expressed genes (DEGs) in IA were identified and the effects of the ICD on immune microenvironment signatures were studied. Techniques like Lasso, Bayes, DT, FDA, GBM, NNET, RG, SVM, LR, and multivariate analysis were used to identify the ICD gene signatures in IA. A consensus clustering algorithm was used for conducting the unsupervised cluster analysis of the ICD patterns in IA. Furthermore, enrichment analysis was carried out for investigating the various immune responses and other functional pathways. Along with functional annotation, the weighted gene co-expression network analysis (WGCNA), protein-protein interaction (PPI) network and module construction, identification of the hub gene, and co-expression analysis were also carried out.

**Results:**

The above techniques were used for establishing the ICD gene signatures of HMGB1, HMGN1, IL33, BCL2, HSPA4, PANX1, TLR9, CLEC7A, and NLRP3 that could easily distinguish IA from normal samples. The unsupervised cluster analysis helped in identifying three ICD gene patterns in different datasets. Gene enrichment analysis revealed that the IA samples showed many differences in pathways such as the cytokine-cytokine receptor interaction, regulation of actin cytoskeleton, chemokine signaling pathway, NOD-like receptor signaling pathway, viral protein interaction with the cytokines and cytokine receptors, and a few other signaling pathways compared to normal samples. In addition, the three ICD modification modes showed obvious differences in their immune microenvironment and the biological function pathways. Eight ICD-regulators were identified and showed meaningful associations with IA, suggesting they could severe as potential prognostic biomarkers.

**Conclusions:**

A new gene signature for IA based on ICD features was created. This signature shows that the ICD pattern and the immune microenvironment are closely related to IA and provide a basis for optimizing risk monitoring, clinical decision-making, and developing novel treatment strategies for patients with IA.

## Introduction

Intracranial Aneurysm (IA) is a highly prevailing life-threatening disease, with a prevalence of 3.2% in adults, worldwide. IA is an abnormal expansion or dilation of intracranial blood vessels in the brain, usually located in the branch of the intracranial artery (1). Rupture of an IA can lead to aneurysmal subarachnoid hemorrhage (aSAH), which is a severe form of stroke (2). Around 30% of IA patients die due to aSAH, and a large majority who survive the stroke cannot carry out regular daily activities (3). Available treatments for IA include surgical treatment, including intracranial aneurysm neck clipping, aneurysm wrapping, and interventional endovascular treatment, like coiling and flow-divert (4–6). However, various risks and complications are associated with treating ruptured or unruptured IA (7). Therefore, identifying IA with high rupture risk and providing timely intervention may be essential for improving risk assessment and treatment.

The primary histopathological features of IA include cell death, immune infiltration, lipid metabolism, oxidative stress, proteolytic activity, and iron accumulation (8). Cell death affects the formation and development of IA and, to a large extent a key component of the IA pathophysiology (9, 10). Immunogenic cell death (ICD) has been identified as a type of regulatory cell death mode (RCD) that triggers an adaptive immune response induced by necrosis or programmed death. This process promotes the maturation of dendritic cells (DC), which present antigens to cytotoxic T cells (CTL), thereby activating CTL to remove adjacent cells and trigger innate and adaptive immune responses (11). In the last few years, many comprehensive research studies were carried out for understanding the mechanism associated with ICD. Damage-related molecular patterns (DAMPs), including the secretion of ATPs, high mobility group protein B1 (HMGB1) release, and surface exposure to calreticulin (CRT), are the key modulators of ICD immunogenicity (12). The mechanism underlying the IA regulation by immunogenic cell death could have potential therapeutic applications in treating unruptured aneurysms. However, previous studies have detected only a few immune cells and immune-related molecules in IA, lacking a view of the ICD in IA.

There is a pressing need to find new biomarkers which could help in the early clinical diagnosis (13, 14) and prognosis (14, 15) of IA patients and explore the potential mechanism of IA progression, which will aid in developing new treatment strategies. Some ICD regulators have been used as typical biomarkers for other diseases. For example, mutations in CALR are widespread in tumors, and CALR interacts with different genes and various proteins (16). HMGB1 can serve as a unique biomarker as well as a target of new therapy in many inflammatory skin diseases (17). Despite the fact that there is mounting evidence that ICD regulates the immune response, these studies do not focus on the role of ICD in the pathogenesis of IA (18). Hence an in-depth investigation of the different immune profiling between the normal tissues and intracranial aneurysm specimens, along with various subtypes of intracranial aneurysm, would help elucidate the changes that occur in ICD and its related genes. This will shed new light on our understanding of the pathogenesis of the intracranial aneurysm.

This study presents a systematic assessment of the ICD-related biomarkers in IA. The results showed that the ICD-regulators could successfully distinguish normal samples from IA samples. Many infiltrating immune cells as well as the immune response gene sets in IA showed a significant correlation with the ICD-regulators. Thus, it was concluded that the ICD-regulators were closely related to immune regulatory factors. Here, IA samples were collected using 22 ICD regulators and three different ICD modification modes were identified. As these subtypes displayed different immune features, their biological functions were compared. The findings of this study indicated that the ICD modification mode significantly impacts the immune microenvironment of intracranial aneurysms.

## Materials and methods

### Intracranial aneurysm datasets and pre-processing

Five independent IA datasets were downloaded from the Gene Expression Omnibus database (GEO database, http://www.ncbi.nlm.nih.gov/geo/), which included GSE15629, GSE13353, GSE75436, GSE26969, and GSE54083. The five gene sets were used as screening sets, and the batch effect from the original data was removed using the sva package (**Figure S1**). A total of 64 IA and 33 normal samples were included in this study. In addition, the GSE122897 dataset was retrieved from GEO and used as a validation set, which included 44 IA samples and 16 normal samples. All the samples were taken from the same tissue type, and the detailed clinical features and platform files of patients are shown in **Supplement file 1**. As per the previous literature, 28 ICD-regulators in the final standardized data set were annotated: *CALR, HMGB1, HMGN1, IL1A, IL33, ROCK1, PANX1, BCL2, PPIA, HSPA4, HSP90AA1, TLR2, TLR3, TLR4, TLR7, TLR9, CLEC4E. CLEC7A, NLRP3, DDX58, IFIH1, AIM2, AGER, TREM1, FPR1, FPR2, CASR, P2R* (18).

### Differences in ICD regulators between different samples and correlation analysis

The differences present in the expression of the ICD-regulators between IA and normal samples were compared with the aid of the Wilcox test. The correlation between the expression of ICD-regulators in the IA and normal samples were also examined using Spearman’s rank correlation analysis.

### Filtering of core ICD regulators using machine learning

The 10-fold Least Absolute Shrinkage and Selection Operator (LASSO) regression method was used for eliminating the redundant genes from 28 gene regulators, and the resulting genes were the core ICD regulators. Based on removed redundant genes, a variety of machine learning models were constructed: Bayes, Decision Tree (DT), Force Directed Algorithm (FDA), Gradient Boosting Machine (GBM), Support Vector Machine (SVM), Neural Network (NNET), RG, and Logistic Regression (LR). Furthermore, the values of the area under the ROC curve (AUC) presented by different models were compared. Finally, LR was determined as the best model. The multi-factor LR was used to calculate the corresponding coefficients of each ICD-regulators, and the final score of each sample (risk score) was obtained.

### Identification and evaluation of nomogram of intracranial aneurysm

The rms package was used to draw the line graphs. Calibration curve, risk decision curve analysis (DCA), AUC, and clinical impact curves were used to evaluate the discrimination performance of scores.

### Identification of ICD pattern

The unsupervised clustering analysis technique was implemented for identifying the varying ICD patterns depending on the core ICD-regulator expression. The consensus clustering algorithm was used for evaluating the number of clusters and robustness. To ensure clustering stability, the k-means clustering method was used to perform 100 iterations (wherein 80% of the samples were used in every run). The optimal number of clustering was determined by determining the clustering score for the cumulative distribution function (CDF) curve. Principal Component Analysis (PCA) validated the reliability of the consensus clustering.

### Differences in immune characteristics and correlation analysis

A single-sample Gene Set Enrichment Analysis (ssGSEA) technique was implemented for determining the number of specific infiltrating immune cells and the immune response activities. ssGSEA also explored the state of immune cells and the immune-linked pathways according to gene sets. A comparison of the enrichment scores of the different immune cells and immune-linked pathways between different ICD modification modes was carried out using the Kruskal-Wallis test. Additionally, the relationship between the core ICD regulators and the immune cells, immune response activity, and HLA expression was determined using Spearman’s rank correlation analysis.

### Gene enrichment analysis for distinct ICD patterns

The “c2.cp.kegg.v7.4.symbols” gene set was downloaded from the MSigDB database and used to study the changes in the biological signaling pathways. The expression matrix was transformed into the score matrix by the gene set variation analysis (GSVA) technique. The score of biological signaling pathways across various ICD modes was compared using the linear models for microarray data (Limma) tool. A statistically significant difference was set at P-values<0.05.

### Identification of the differentially expressed genes noted between different ICD patterns

The Limma package screened DEGs between different ICD patterns. P<0.05 was used as a screening standard. The Gene Ontology (GO) and the Kyoto Encyclopedia of Genes and Genomes (KEGG) pathway enrichment analyses were carried out using the clusterProfiler package.

### Weighted gene co-expression network analysis

The WGCNA R software package was applied for evaluating the training set for the first 25% variance of the gene matrix. The construction of the WGCNA network and the module detection were done using a sign-less Topology Overlap Matrix (TOM). The optimal soft threshold value was recorded to be 3, while a minimum of 50 genes were included in the module, with a truncation height of 0.2. The relationship between the merged modules and the various ICD modification modes was determined by performing Spearman’s rank correlation analysis. Finally, the core protein in the module was defined as the top ten MCC genes present in the protein-protein interaction (PPI) network. Cytoscape 3.7.1 was used for visualization.

## Results

### Expression landscape of ICD-regulators among different samples

**Figure 1:**
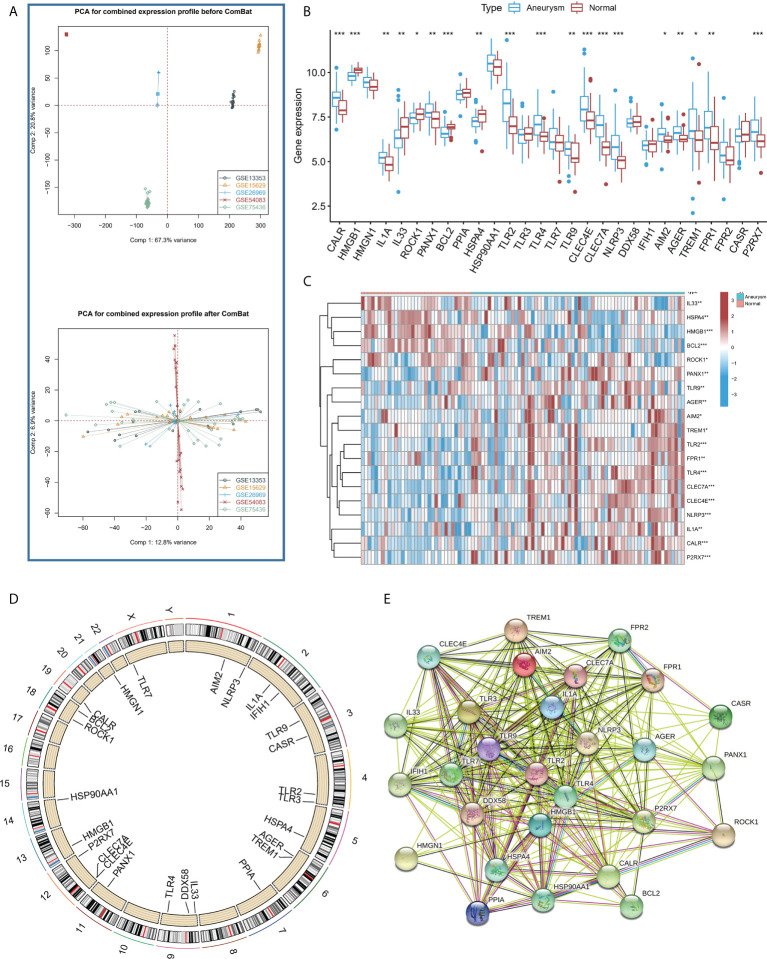
**(A)** PCA results for the combined expression profile before Combat and after Combat. **(B)** The box plot demonstrated the transcriptome expression status of 28 immunogenic cell death (ICD) regulators between intracranial aneurysms (IA) and normal samples. **(C)** The heatmap plot showed significant differences in terms of the expression levels of 19 regulators between IA and normal samples. **(D)**. The landscape of gene expression changes of ICD-regulators in the IA. The location of the CNV alteration of ICD-regulators on the 22 chromosomes derived from the GEO database. **(E)** The composition summary of the ICD-regulators and PPI among the 28 ICD-regulators. *P < 0.05, **P < 0.01, ***P < 0.001.

**Figure 1A** shows that the combat algorithm can better remove the batch effect between different datasets. There were 28 ICD regulators involved in the study, and the correlation expression of different regulators was analyzed in normal (**Figure S1A**) and IA samples (**Figure S1B**). The results revealed that the TLR2 and most regulators showed a strong positive correlation with all the samples. In addition, the Wilcox test results highlighted the significant variations in the expression levels of 19 regulators between the IA and normal samples (**Figures 1B, C**). The location of the 19 regulators on chromosomes is shown in **Figure 1D**. In addition, the regulatory interactions of these ICD-regulators were manifested as PPI networks. It was found that the different regulators were very closely linked and usually functioned as a complex (**Figure 1E**). In addition, we used the Enrichr databased to predict therapeutic drug based ICD regulators, and we found therapeutic drug maybe Hydroxychloroquine, Hydroxy Radical Formation Stimulant, etc (**Supplemental Files 2**).

### ICD-regulators as potential biomarkers for intracranial aneurysm

**Figure 2:**
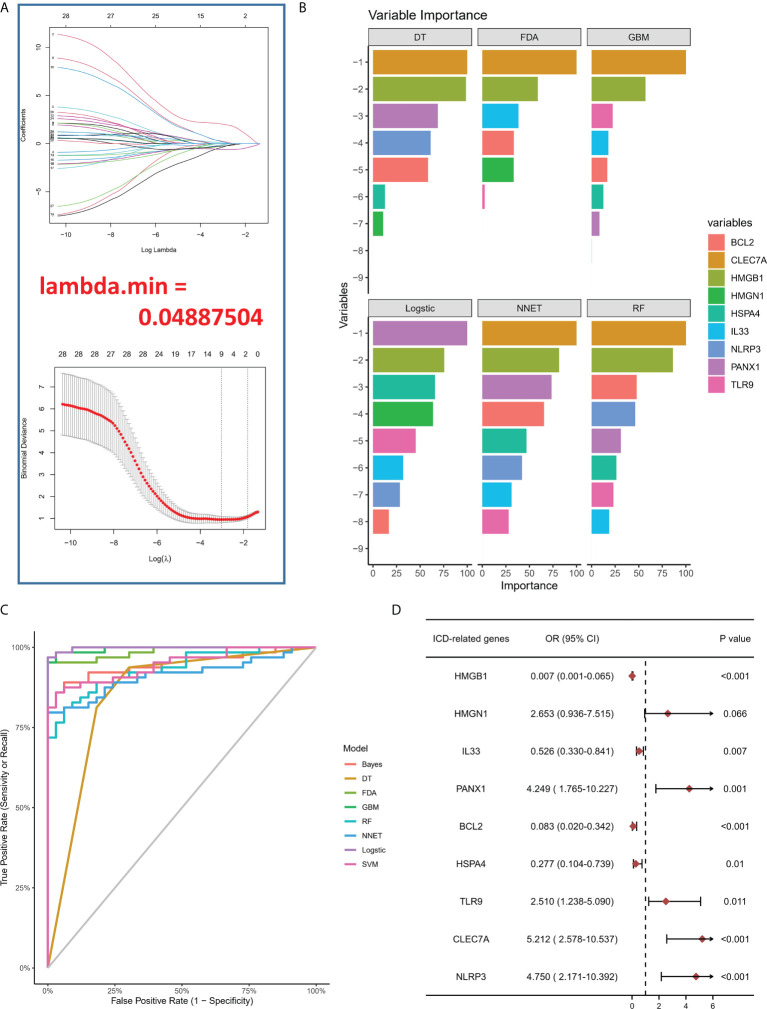
**(A)** LASSO coefficient profiles of IA-linked ICD-regulators. 10-fold cross‐validation for tuning parameters using the LASSO regression. **(B)** Variable importance plot in machine learning. **(C)** ROC curves show the AUC values of various machine learning models in model comparison. **(D)** Logistic regression highlighted the correlation between ICD-regulators and IA samples, which yielded nine IA-linked ICD-regulators (P <0.05).

**Figure 3:**
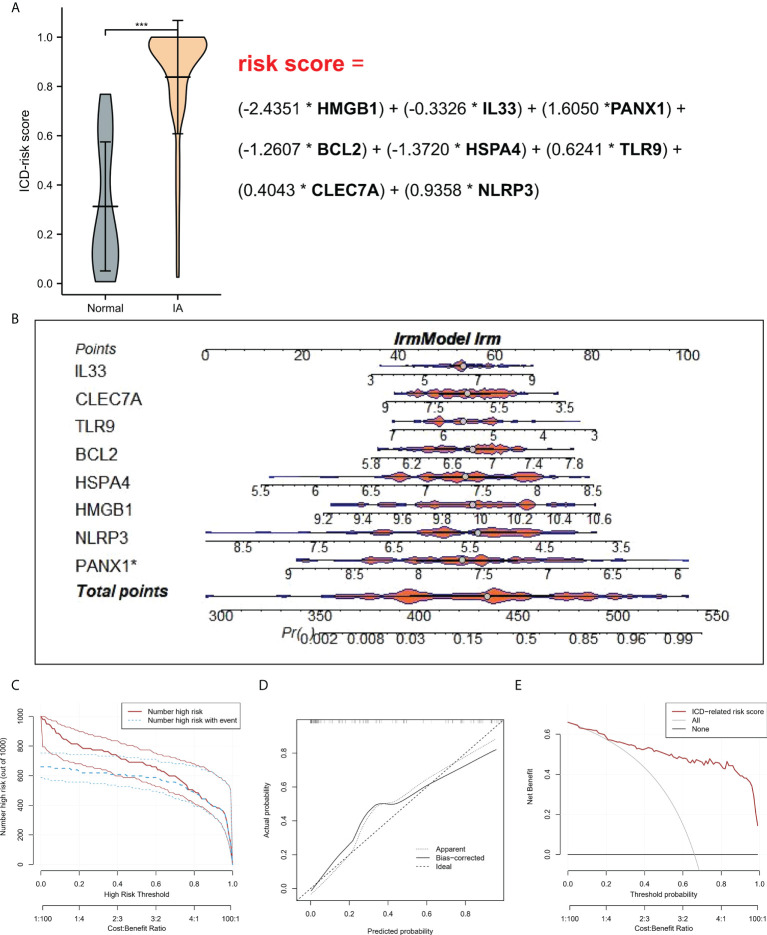
**(A)** Risk distribution between IA and normal samples, where IA showed a higher risk score compared to the normal samples. **(B)** Nomograms for predicting the risk scores of nine IA-related ICD-regulators. **(C)** Every variable was given a score, where the total was transformed into a probability on the model’s lowest scale of the clinical impact curves. **(D)** Plots show the model-related calibration in terms of the consistency between predictions. In the nomogram, Y-axis presents the observed IA, while the X-axis presents the estimated IA. **(E)** Decision curves for risk prediction models for IA. The vertical axis depicts the net benefit of standardization. The two horizontal axes present the correlation between the cost-benefit ratio and the risk threshold. * refers to multiplication. ***P < 0.001.

To study the contribution of ICD-regulators in the pathogenesis of IA, LASSO regression was carried out on 28 regulators for feature selection, while the dimensionality reduction procedure was implemented for eliminating the redundant genes (**Figure 2A**). Finally, nine genes were selected and used for subsequent analysis. Subsequently, the machine learning models of Bayes, DT, FDA, GBM, NNET, RG, SVM, and LR were used to determine the value of core regulators in diagnosing IA. **Figure 2B** shows the importance plot of nine genes in different models. As shown in **Figure 2C**, the LR model had the best AUC value in the model comparison. Subsequently, the OR values of nine genes for the occurrence in IA samples have been presented. HMGB1, IL33, BCL2, and HSPA4 were protective factors, while PANX1, TLR9, CLEC7A, and NLRP3 were risk factors (**Figure 2D**) for IA. Finally, the multi-factor LR model was used to calculate the final risk score. The final risk score = (-2.4351 * HMGB1) + (-0.3326 * IL33) + (1.6050 *PANX1) + (-1.2607 * BCL2) + (-1.3720 * HSPA4) + (0.6241 * TLR9) + (0.4043 * CLEC7A) + (0.9358 * NLRP3). The classifier consisted of nine ICD regulators, wherein the risk scores of IA samples were significantly higher compared to those of the normal tissue samples (**Figure 3A**).

### Construction of nomogram model

**Figure 4:**
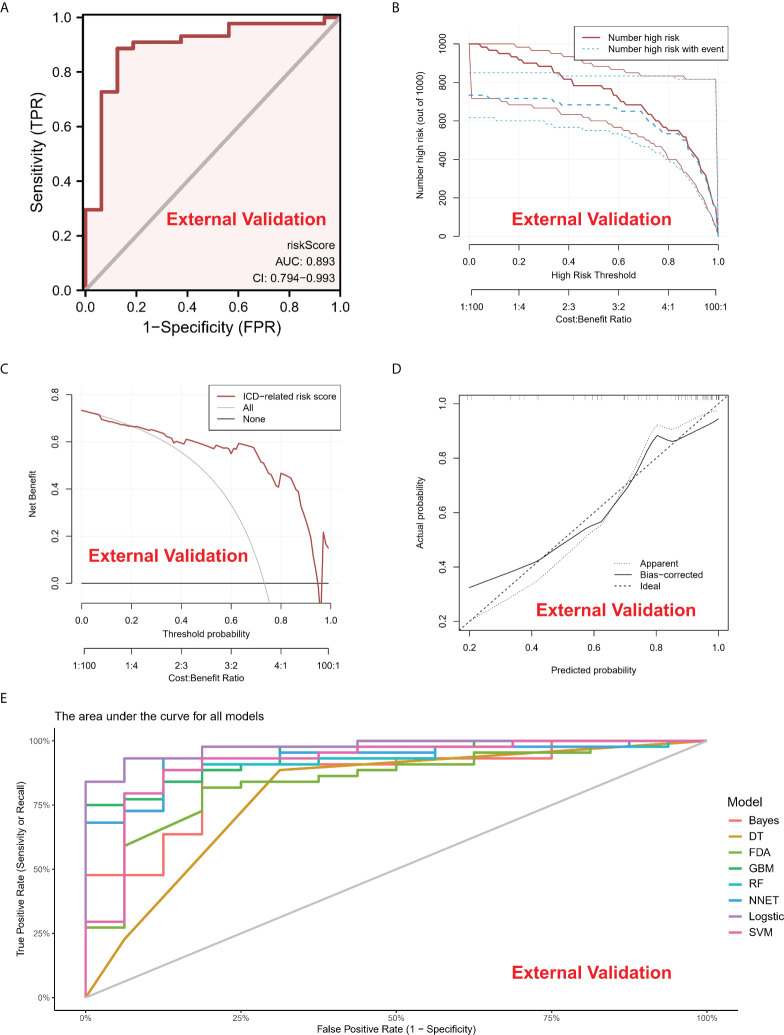
**(A)** In the External Validation cohort, the ROC curve and AUC value were used to study and assess the capacity of ICD-regulators to distinguish between normal and IA samples. **(B–D)**. The clinical impact, decision, and calibration curves for the model in the external validation dataset. **(E)** AUC values of all models in the External Validation dataset.

A line diagram model based on eight ICD regulators was constructed (**Figure 3B**). In the training set, the clinical influence curve revealed that the line graph model showed a remarkable predictive ability (**Figure 3C**). Furthermore, the calibration curve indicated that the line graph model showed an accurate predictive ability (**Figure 3D**). In the DCA curve, the red line was always above the gray line, indicating that the decision based on the line graph model can benefit IA patients (**Figure 3E**). In the validation set, the risk score showed an AUC value of 0.893 (**Figure 4A**). The clinical influence, calibration, and DCA curves also showed a strong diagnostic prediction ability (**Figures 4B–E**).

### ICD-regulators mediated patterns in intracranial aneurysm

**Figure 5:**
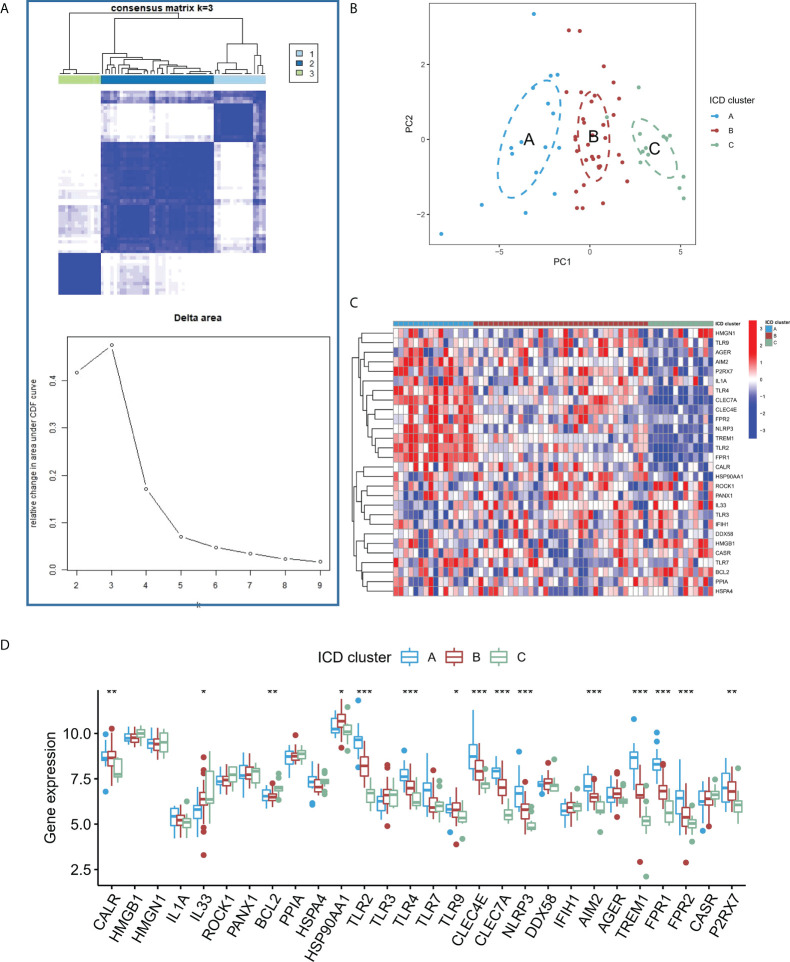
**(A)** Heatmap describing the co-occurrence proportion matrix for IA samples and the relative changes in the area under the CDF curve for k = 2–9. **(B)** Results of PCA of the transcriptome profiles of three ICD subtypes that exhibit differences in the transcriptome across various patterns. **(C)** Results of the unsupervised clustering analysis of 28 ICD-regulators determined in the three patterns. **(D)** Expression levels of the 28 ICD regulators in the three ICD subtypes. *P < 0.05, **P < 0.01, ***P < 0.001.

Based on the expression of the core regulators, an unsupervised consistent cluster analysis was performed on 63 IA samples, and three different ICD modification subtypes were identified (**Figure 5A**). PCA analysis showed that IA patients can be further categorized into three groups based on the ICD-regulators (**Figure 5B**). Significant differences were observed in the expression of some ICD-regulators among different modification modes (**Figures 5C, D**). In addition, we conducted unsupervised consistent cluster analysis in external dataset, and significant DEG were similar with modeling dataset (**Figure S2**).

### Identification of immune microenvironment and biological function characteristics in different ICD patterns

**Figure 6:**
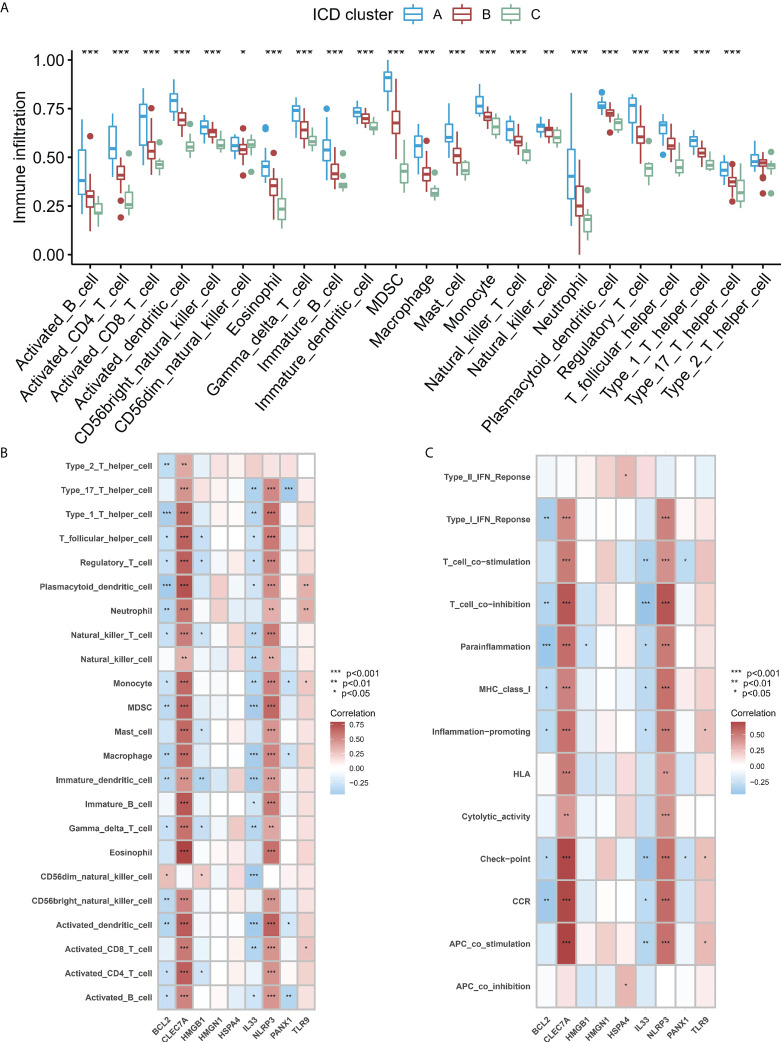
**(A)** Significant differences were noted in each type of immune microenvironment -infiltrating immune cells in the three ICD patterns. **(B, C)**. Correlation between the immune reaction gene-sets, infiltrating immunocytes, and ICD-regulators. **(B)** The square plot highlighted the relationship between every dysregulated immune microenvironment infiltration cell type and each dysregulated ICD-regulators **(C)** Square plot highlighting the relationship between every dysregulated immune response gene set and every dysregulated ICD-regulators. *P < 0.05, **P < 0.01, ***P < 0.001.

To study the difference in the immune microenvironment features between the different ICD models, the differences in the infiltrating immune cells and their immune functions were analyzed. Compared to models B and C, model A has relatively higher activation of T cells and Natural Killer (NK) cells (**Figure 6A**). Regarding the immune responses, model A’s immune response was more active (**Figure S3A**) than models B and C. In addition, the expression of different HLA was also different among the modified models (**Figure S3B**). The expression of HLA in model A was higher than in models B and C. These results again validated that ICD modification plays an important regulatory role in forming different immune microenvironments in IA patients. In addition, to study the biological behavior between ICD-regulators and the immune microenvironment, the correlation between the nine core regulators and the expression levels of infiltrating immune cells and immune-linked pathways, was analyzed. The results showed that nine core regulators were closely associated with many immune cells in IA samples (**Figure 6B**), especially CLEC7 A and NLRP3 positively correlated with most immune cells in IA samples. In contrast, BCL2 and IL33 negatively correlated with most immune cells in IA samples. In terms of immune function, the results showed a close correlation between immune cells in IA samples (**Figure 6C**). These results show the role of the core ICD-regulator in the IA immune microenvironment.

### Biological properties of different ICD patterns

**Figure 7:**
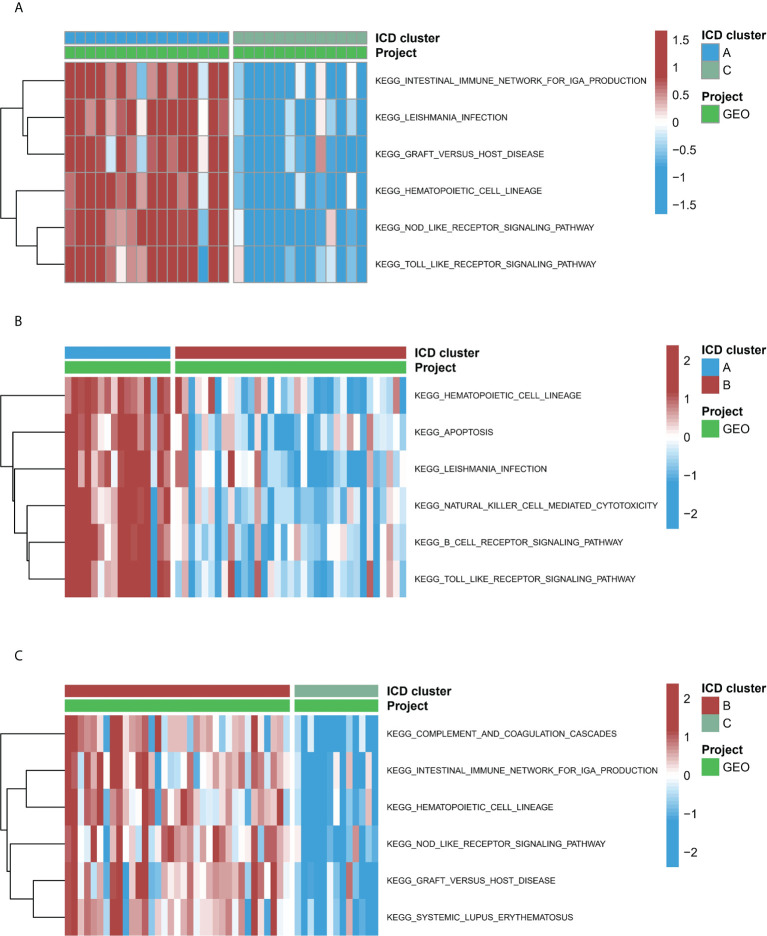
The underlying biological function characteristics diversity across three ICD patterns. **(A)** The variations in KEGG pathway enrichment scores across ICD patterns A and C. **(B)** The variations in KEGG pathway enrichment scores across ICD patterns A and B. **(C)** The variations in KEGG pathway enrichment scores across ICD patterns B and C.

**Figure 8:**
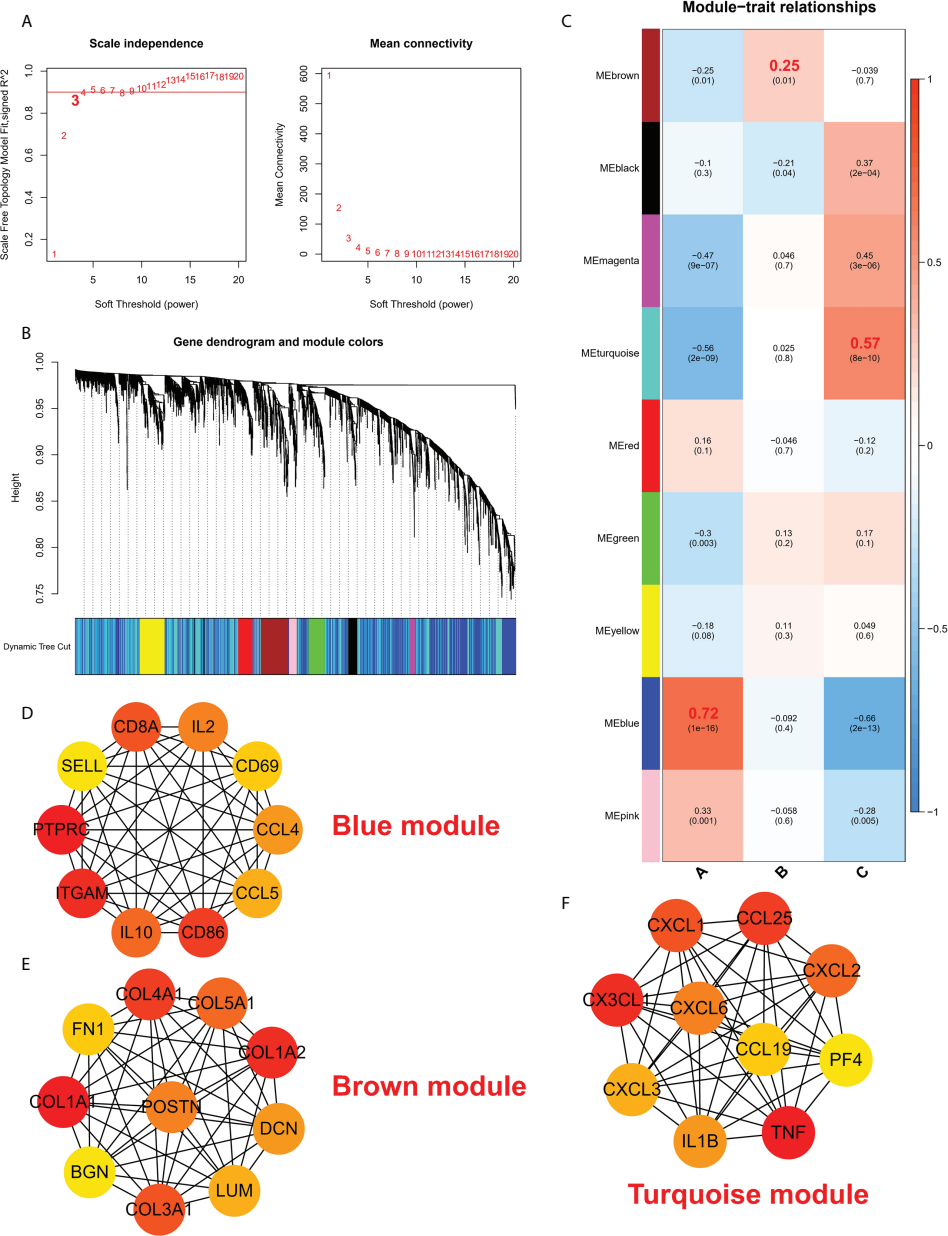
**(A)** Analysis of a scale-free ft index and mean connectivity for different soft-thresholding powers. **(B)** Gene dendrogram was developed using average linkage hierarchical clustering. The module assignment decided by the Dynamic Tree Cut, in which nine modules were discovered, is displayed in the color row beneath the dendrogram. **(C)** A heatmap showing how module eigengenes and ICD patterns are correlated. d-f. Hub protein-related PPI networks in the blue module **(D)**, brown module **(E)**, and turquoise module **(F)**.

To study the biological functions in the three ICD models, the KEGG pathways were compared. Furthermore, the GSVA enrichment analysis was implemented to evaluate the activation of the biological signaling pathways in the three ICD models. The results indicated that in comparison to model C, the intestinal immune network for the NOD-like receptor, IgA production, and the TOLL-like receptor signaling pathways (**Figure 7A**) were remarkably enriched in model A. Furthermore, compared to Model B, the NK cell-mediated toxic signaling pathway and the B-cell receptor signaling pathway were remarkably enriched in model A (**Figure 7B**). As shown in **Figure 7C**, the complement and coagulation cascades signaling pathway was remarkably enriched in model A, compared to model B. In addition, 664 DEGs (**Supplementary Files 2**) were identified in different modification modes. Further enrichment analysis was carried out using these DEGs. GO enrichment analysis results showed these genes were mainly involved in various mechanisms such as lymphocyte activation, inflammatory response regulation, innate immune response regulation, T cell activation, and neutrophil degranulation (**Figures S4A–C**). Using the KEGG enrichment analysis, pathways like NOD-like receptor signaling, regulation of actin, and cytokine-cytokine receptor interaction were screened out. The interaction between the viral proteins and cytokine receptor pathways was significantly associated with the IA samples (**Figure S4D**). The WGCNA method was used to determine the gene-gene modules linked to various ICDs models (**Figures 8A, B**). Three key gene modules were identified as associated with different ICD models (**Figure 8C**). The blue module was associated with subtype A (r = 0.72), the brown module was associated with subtype B (r = -0.25), and the blue module was associated with subtype C (r = 0.57).

### Characterization of coregulatory proteins with different ICD patterns

The protein interaction network of the brown module, grey module, and red module was established with the help of the STRING database, and then, the MCC values of each protein were calculated using Cytoscape. The results showed that subtype A may be mainly regulated by *CD8A, IL2, CD69, CCL4, CCL5, CD86, IL10, ITGAM, PTPRC*, and *SELL* (**Figure 8D**); Subtype B may be mainly regulated by *COL4A1, COL5A1, COL1A2, DCM, LUM, COL3A1, BGN, COL1A1, FN1, POSTN* (**Figure 8E**); Subtype C may be mainly regulated by *CXCL3, CX3CL1, CXCL1, CCL25, CXCL2, PF4, TNF, IL1B, CXCL6 and CCL19* (**Figure 8F**).

## Discussion

Different types of cell death processes, like apoptosis, necrotizing, autophagy, ferroptosis, copper-induced cell death, non-procedure necrosis, and immunogenic cell death, have been widely studied in many diseases and have led to the development of many therapeutic modalities (19, 20). Many studies confirmed that ICD was significantly involved in the pathogenesis of multiple diseases, such as cancers (21). In the present study, significant differences were noted in the expression of most ICD-regulators between the normal and IA samples. Lasso regression, multiple machine learning models (Bayes, DT, FDA, GBM, NNET, RG, SVM, LR), and multiple factors LR were used to determine ICD-regulators gene patterns, including *HMGB1, HMGN1, IL33, BCL2, HSPA4, PANX1, TLR9, CLEC7A, NLRP3*. IA and normal samples can be easily distinguished, emphasizing the difference in the ICD gene signatures between the two.

Among the 28 ICD-regulators studied using LASSO, nice ICD- regulators were selected for subsequent analysis. Many ICD-regulators have extensive protein interactions or expression correlations, revealing the extensive regulatory network of ICD modifications. The correlation analysis among ICD-regulators and immune characteristics of IA was studied, and different immune response gene sets, infiltrating immune cells, and HLA gene expression were carried out. The results suggest that a wide range of ICD-regulators were strongly linked to these immunological features, indicating that ICD is crucial in modulating the immune microenvironment of IA.

Based on core ICD-regulator expression profiles and unsupervised clustering analysis, ICD patterns in IA were studied, and three subtypes were identified with unique ICD patterns. Each subtype has particular immunological traits of its own. The modified model A has a higher infiltration level of immune cells than models B and C, indicating a more active immunological response. The immunological traits of each subtype validated the robustness of the classification of various ICD-regulators used in this study. The classification method of immune subtypes helps elucidate the potential mechanism of immune regulation. To develop accurate treatment strategies, IA subtypes could further be analyzed at the molecular or immune level rather than the phenotypic characterization. A recent study used a similar method to identify two different ICD patterns in the head and neck squamous cell carcinoma, which enhanced the understanding of the tumor microenvironment and helped develop a more effective immunotherapy strategy (22). Lu et al. used WGCNA and ssGSEA methods to analyze IA and normal samples and identified immune-related genes that mediate IA. The final results showed that inflammation and immune responses are involved in IA pathogenesis (23).

Here, different patterns of ICD-regulators and ICD gene signatures were identified. The expression and regulation of these genes are affected by ICD. Understanding their biological functions may help understand the role played by ICDs in IA pathogenesis. Additionally, from the functional pathway perspective, compared to model C, model A greatly enriched the intestinal immune network for the signaling pathways that control IgA synthesis, NOD-like receptor signaling, and TOLL-like receptor signaling. When compared to model B, the natural killer cell-mediated toxic signaling pathway and B cell receptor signaling pathway were enriched more in model A. Compared to model B, the complement and coagulation cascade signaling pathway in model C was significantly enriched. Finally, nine ICD regulators were determined. HMGB1, IL33, BCL2, and HSPA4 were protective factors, while PANX1, TLR9, CLEC7 A, and NLRP3 were risk factors for IA. No significant differences were noted in the HMGN1 expression levels between the IA and normal samples.

HMGB1 is a high mobility group B1 protein is a nonhistone chromatin-binding protein. It regulates transcription and DNA repair and is remarkably involved in many cellular processes, including immune responses, inflammation, cell proliferation, cell differentiation, and cell migration (24). Recent studies have shown that association between RAG/MR/HMGB1 and ATP1α3 imbalance and inflammatory changes in vulnerable cerebral aneurysms (25). Zhang et al. demonstrated the expression of HMGB1 in brain aneurysms; there was higher HMGB1 expression in ruptured aneurysm tissue compared to unruptured tissues aneurysm tissue (26). amRF promotes the expression of the heat shock protein 70 (HSP70) on the plasma membrane, which participates in the ICD pathway, and guides the sequential molecular changes that trigger innate and adaptive immune responses. Extracellular HSP70 forms damage-related molecular patterns (DAMP) with free HMGB1 and membrane expression of calreticulin (CRT) (27). In addition, Micheliolide (MCL) induces ICD-related DAMP (such as CRT exposure, ATP secretion, and HMGB1 release). Reports suggest in the mouse vaccine model, MCL triggered the regression of tumors by inducing ICD, involving the DCs maturation, stimulation of CD4 and CD8 T cells, and release of HMGB (28). Additionally, photothermal therapy and the CDT-mediated ICD improve anti-tumor immunity by exposing HMGB1, CRT, and ATP. Reports also suggest Amphotericin B (AmB) enhances the anti-tumor immune response by releasing HMGB1, which induces ICD (29). Studies show a correlation between high serum concentration of IL-33 and inflammation, poor prognosis, and severity of aSAH. Elevated serum IL-33 concentration is indicative of a poor prognosis of aneurysmal subarachnoid hemorrhage (30). Thus, suggesting that IL-33 could be considered as a potential inflammatory biomarker that could be used for evaluating the prognosis and severity of aSAH. BCL2 (BCL2 Apoptosis Regulator) is an anti-apoptotic protein that prevents the death of specific cells like lymphocytes. The expression of BCL2 may reflect the enhancement of cell death and immunogenicity risk signals in transplantation and constitute a risk factor for poor transplantation results (31). Further, HSPA4 is expressed in heat resistance, damage signaling molecules (DAMPs), and ICD (32).

ICD represents a special class of apoptosis that triggers an adaptive immune response, in which ATP secretion depends on the molecular mechanism of autophagy. Studies have found that pannexin 1 (PANX1) -dependent lysosome exocytosis mediates ATP release (33, 34). TLR9 (Toll-Like Receptor 9) mediates the production of cytokines required for an effective immune response. It also mediates cell responses to unmethylated CpG dinucleotides in bacterial DNA, thereby initiating innate immune responses. Studies have shown that TLR9 agonists enhance radiofrequency ablation-induced CTL (cytotoxic T lymphocyte) response, effectively inhibiting tumor growth and lung metastasis (35). In addition, studies have shown that intravenous injection of TLR9 agonists nanoparticles can detect tumor microenvironment, generate localized immune activation and release the dead tumor cells into the circulation, which are then taken up by the antigen-presenting cells to trigger an anti-tumor immune reaction by stimulating tumor antigen-specific CD8 T cell (36). In addition, inhaled TLR9 agonists make lung tumors more tolerant to PD-1 blockade by promoting the interaction between CD4 and CD8 T cells (37).

NLRP3 (NLR Family Pyrin Domain Containing 3) contains a nucleotide-binding site domain (pyrin domain) and a leucine-rich repeat motif. It interacts with PYCARD/ASC, an apoptosis-related spotted protein, which contains the caspase recruiting domain and belongs to the NLRP3 inflammasome complex. Studies have shown that tumor cell-derived IL-1β promotes the proliferation of fibrous tissue and immunosuppression in pancreatic cancer. This phenomenon depends on tumor cells activating the NLRP3 inflammasome to produce IL-1β (38). Together these studies indicate that ICD-regulators may induce inflammatory responses, thereby emphasizing the association between ICD-regulators in immune responses and their involvement in regulating immune responses.

However, the study has a few limitations. The clinical data of every patient, like age, gender, Hunt-Hess grade, treatment, and disease prognosis, was unavailable; hence, it could not be analyzed. Further, the datasets were acquired from GEO and had a limited sample size. Therefore, studies with a large sample size are required to thoroughly understand the involvement of ICD in IA. In addition, due to the difficulty of obtaining IA samples, we did not conduct *in vitro* experiments. In summary, this study evaluates the role of ICD in IA patients. The study revealed that ICD-regulators could easily distinguish IA from normal and determined three different ICD subtypes based on 28 ICD-regulators. The three ICD subtypes of IA significantly differed in ICD expression, immune microenvironment, and biological function pathways. A close association was observed between ICD subtypes and immune characteristics. This could have a potential role in the development of targeted immunotherapy. In addition, 8 ICD-regulators were identified that could be used as potential prognostic biomarkers for the treatment of IA.

## Data availability statement

The datasets presented in this study can be found in online repositories. The names of the repository/repositories and accession number(s) can be found in the article/**Supplementary Material**.

## Author contributions

AM and MT: conceptualization, methodology, validation, investigation, supervision, software, visualization, writing - original draft, and writing- reviewing and editing. DG, AA, NR, RS, KK, ZW, XjC, YZ, and MA participated in the coordination of data acquisition and data analysis and reviewed the manuscript. All authors contributed to the article and approved the submitted version.

## Funding

This work was supported by the National Natural Science Foundation of China (grant numbers: 81801158) and Young Scientist Program of BeijingTiantan Hospital Affiliated to Capital Medical University (grant numbers: YSP201904).

## Acknowledgments

We are grateful to the contributors to the public databases used in this study.

## Conflict of interest

The authors declare that the research was conducted in the absence of any commercial or financial relationships that could be construed as a potential conflict of interest.

## Publisher’s note

All claims expressed in this article are solely those of the authors and do not necessarily represent those of their affiliated organizations, or those of the publisher, the editors and the reviewers. Any product that may be evaluated in this article, or claim that may be made by its manufacturer, is not guaranteed or endorsed by the publisher.

## Supplementary material

Supplementary Figure 1
**(A, B)** Relationships between the expression levels of 28 ICD-regulators in IA and normal samples.

Supplementary Figure 2 DEGs in external validation dataset.

Supplementary Figure 3
**(A)** Variations in the expression levels of 13 immune response gene sets between IA and normal tissue samples. **(B)** Variations in the expression levels of 18 HLA genes between IA and the normal samples.

Supplementary Figure 4
**(A–C)** CC, GO-BP, and MF functional enrichment analyses highlight the biological features of the ICD phenotype-linked genes. d. The KEGG enrichment analysis of the ICD phenotype-linked immune genes reveals the correlation between immune regulation and ICD-regulators.
