# Supplementary figures and images for: An immunogenic cell death-related regulators classification patterns and immune microenvironment infiltration characterization in intracranial aneurysm based on machine learning
Source: Front Immunol. 2022 Sep 29;13:1001320. doi: 10.3389/fimmu.2022.1001320 (PMC9556730; doi:10.3389/fimmu.2022.1001320)

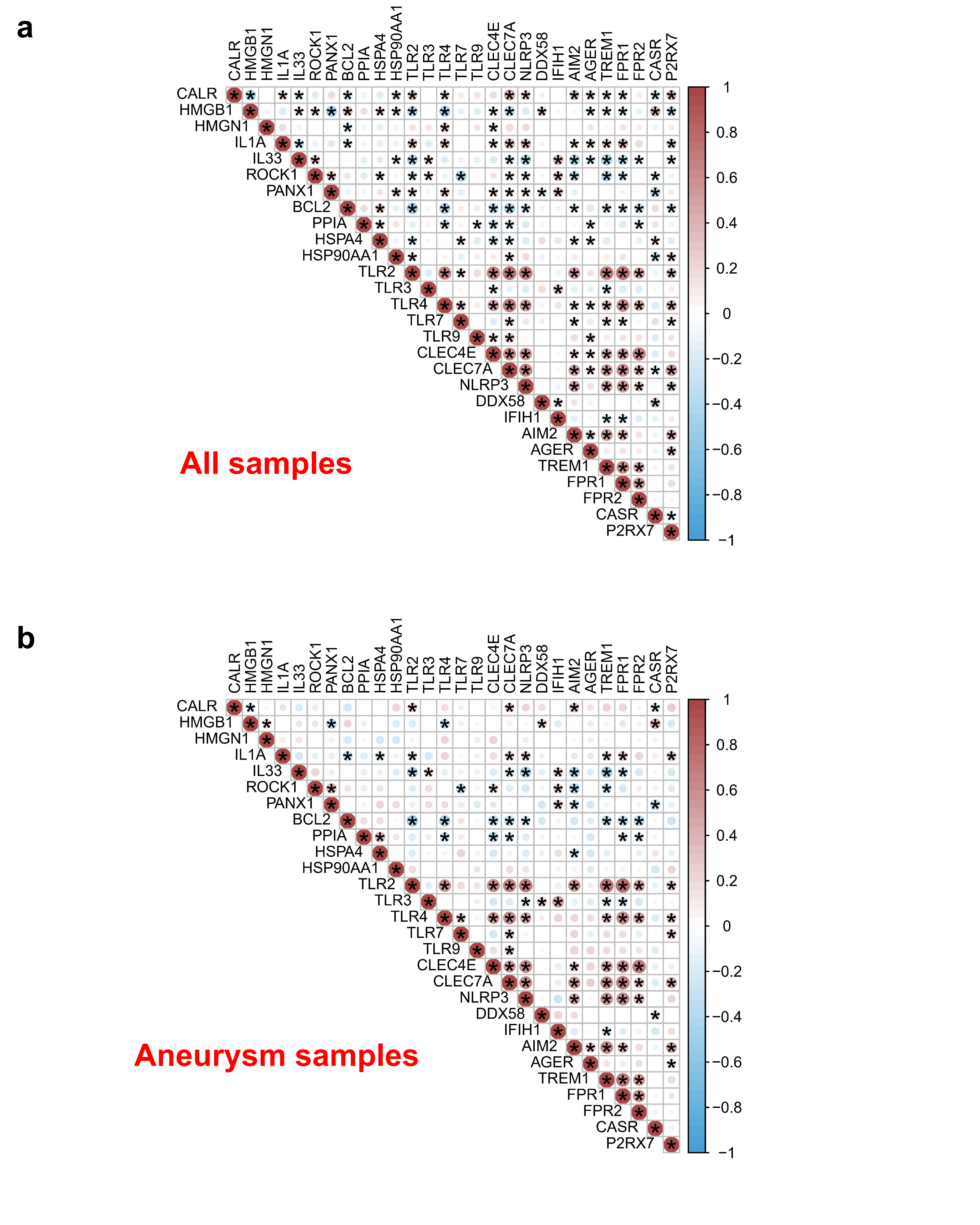

Supplement: Supplementary Figure 1 — (A, B) Relationships between the expression levels of 28 ICD-regulators in IA and normal samples. [file Image_1.tiff]

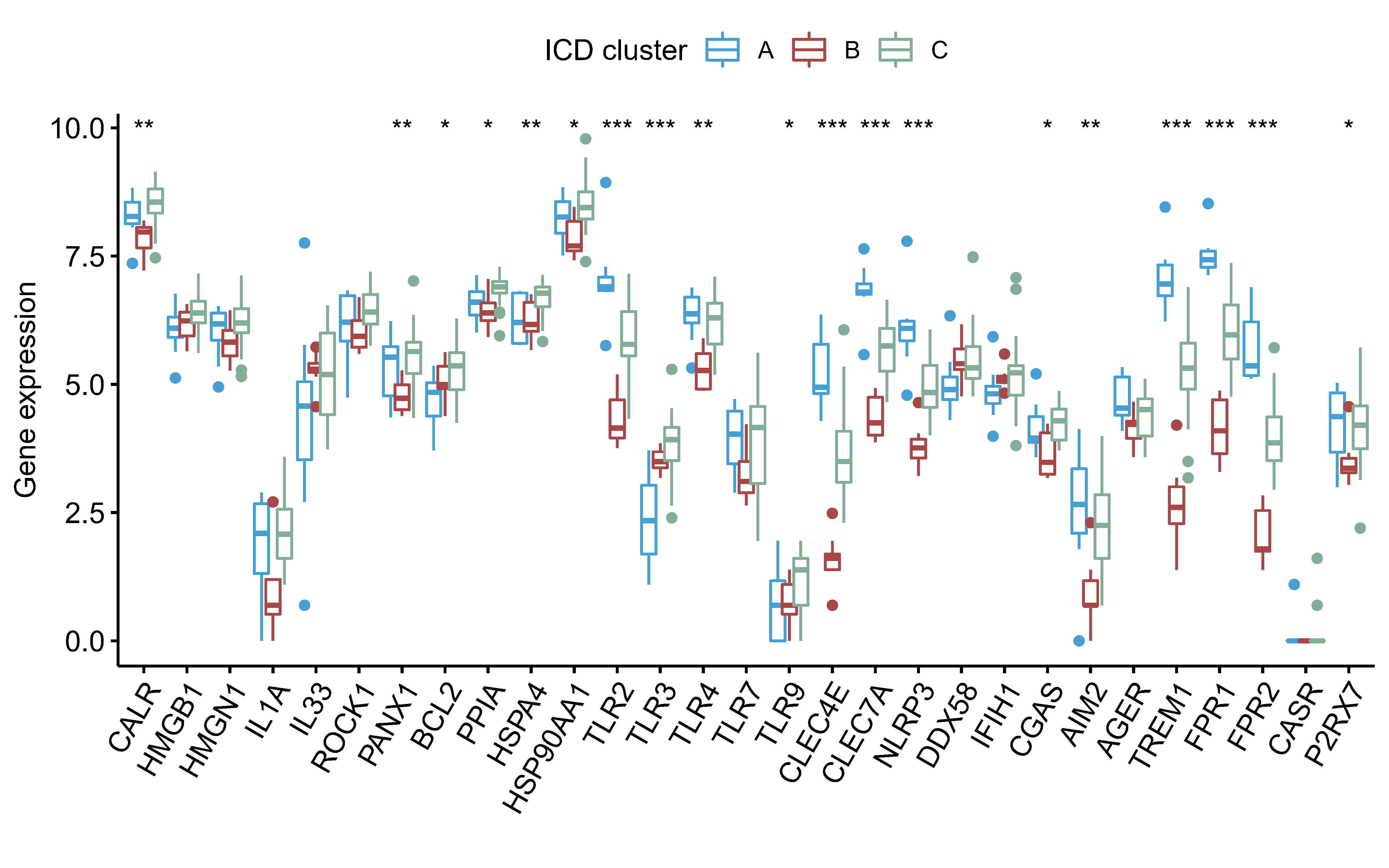

Supplement: Supplementary Figure 2 — DEGs in external validation dataset. [file Image_2.tif]

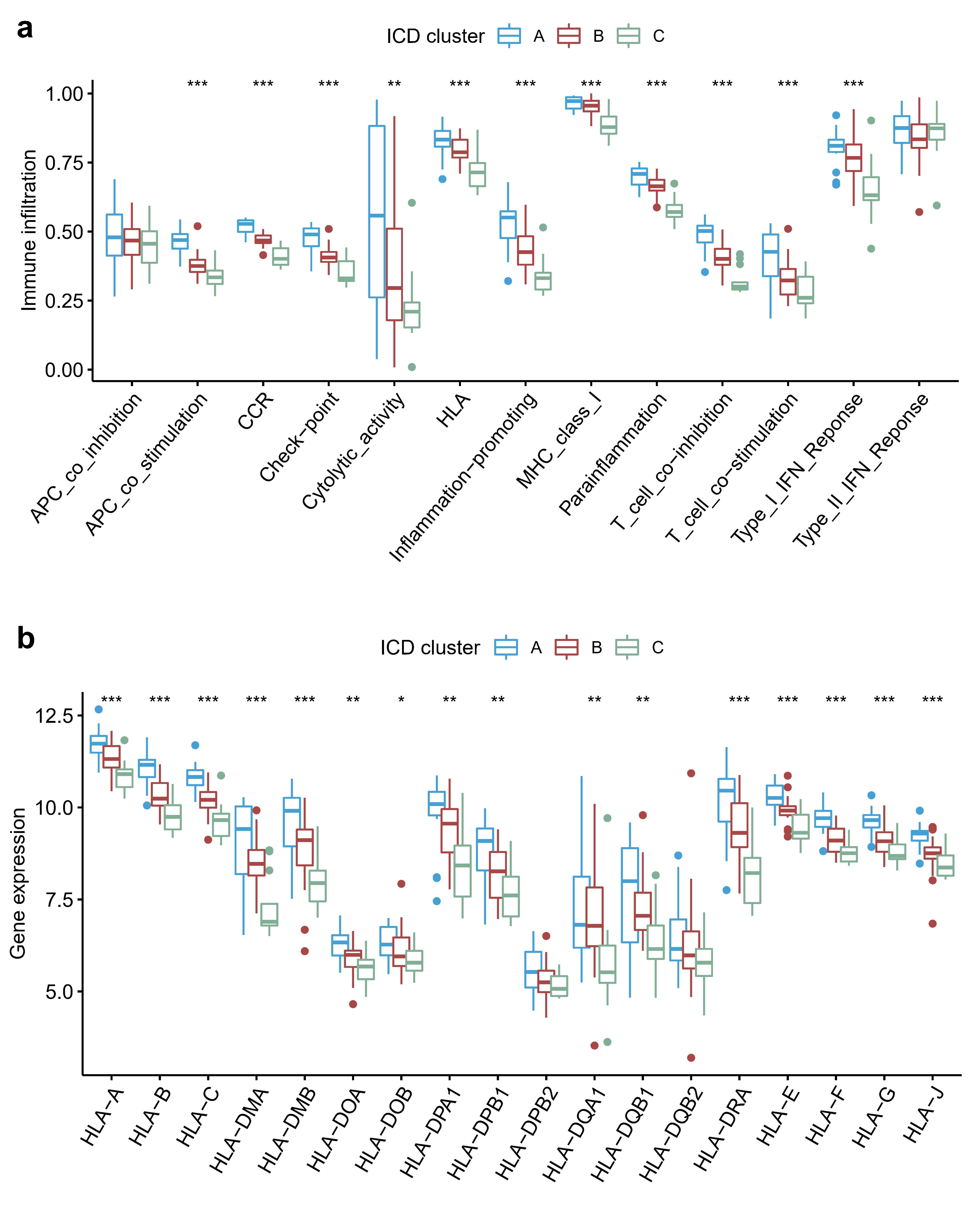

Supplement: Supplementary Figure 3 — (A) Variations in the expression levels of 13 immune response gene sets between IA and normal tissue samples. (B) Variations in the expression levels of 18 HLA genes between IA and the normal samples. [file Image_3.tiff]

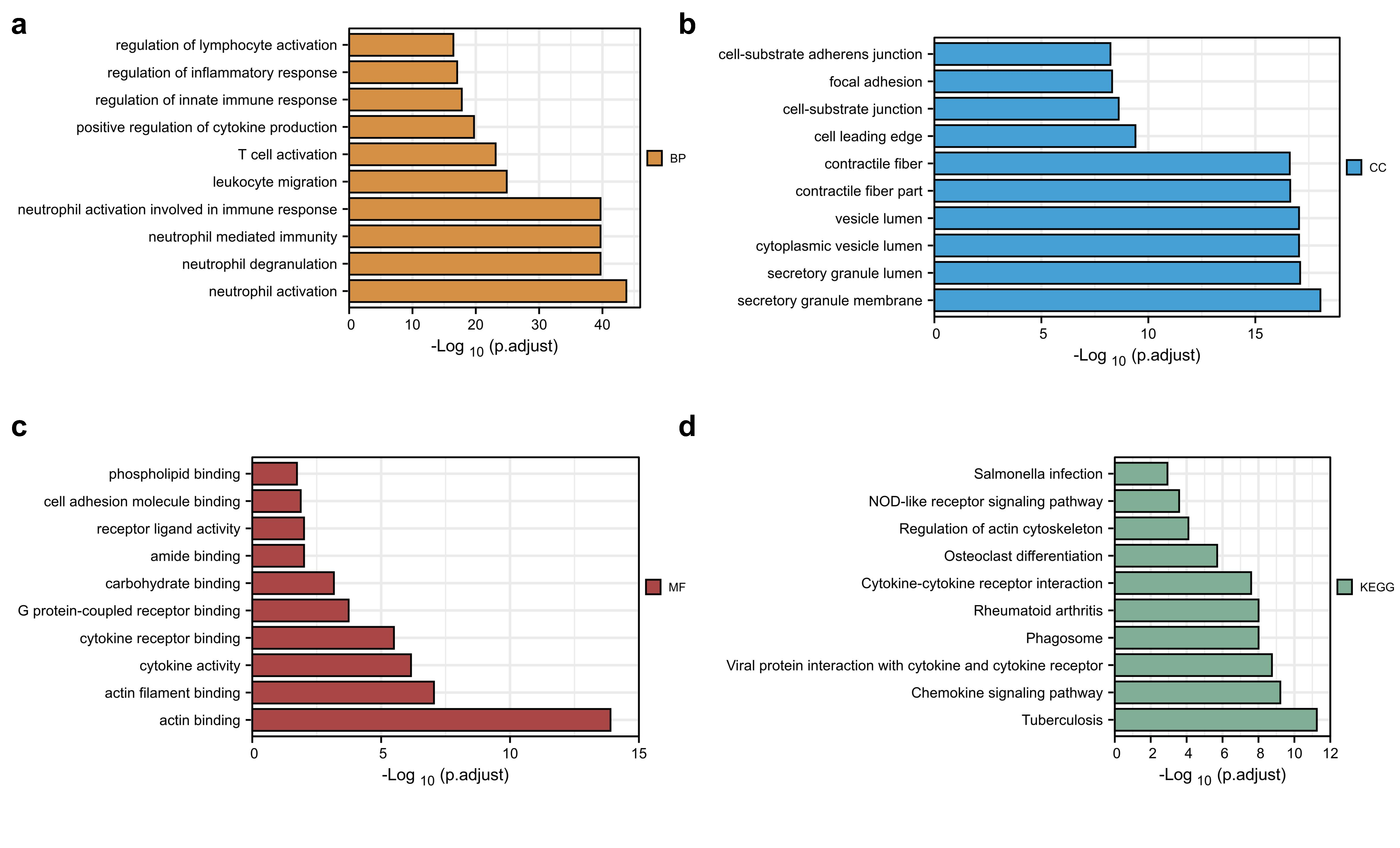

Supplement: Supplementary Figure 4 — (A–C) CC, GO-BP, and MF functional enrichment analyses highlight the biological features of the ICD phenotype-linked genes. d. The KEGG enrichment analysis of the ICD phenotype-linked immune genes reveals the correlation between immune regulation and ICD-regulators. [file Image_4.tiff]
